# 101 Identity leadership in Zumba classes: Does it matter to create a a sense of ‘us’ in temporary exercise groups?

**DOI:** 10.1093/eurpub/ckae114.077

**Published:** 2024-09-26

**Authors:** Filip Boen, Louis Bonte, Quentin Brabant, Thomas Könecke, Sean Figgins, Katrien Fransen

**Affiliations:** KU Leuven, Leuven, Belgium; KU Leuven, Leuven, Belgium; KU Leuven, Leuven, Belgium; KU Leuven, Leuven, Belgium; University of Sussex, Brighton, United Kingdom; KU Leuven, Leuven, Belgium

## Abstract

Inspired by the social identity approach, recent work has emphasized the importance of identity leadership in temporary exercise groups to boost participation and performance. According to this social identity approach, effective identity leaders are those who can encourage their followers to think, feel, and act as part of the group rather than as individuals. To achieve this, identity leaders should be perceived as prototypical for the group (i.e., being one of “us”), as advancers of the group (i.e., acting for “us”), as group entrepreneurs (i.e., crafting a sense of “us”), and as group impresarios (i.e., making “us” matter).

However, while group exercise leaders usually receive extensive training in physiological and biomechanical movement principles, they are often less educated on how to effectively engage a group of exercisers as a cohesive unit. This seems nevertheless very relevant, considering that many of these exercise groups are temporary, with members meeting only a limited number of times and often not knowing each other (well). Moreover, the composition of these exercise groups can change during the course of the classes: new members can attend, while older members attend irregularly or drop-out, which poses an additional challenge to create a sense of ‘us’.

In a cross-sectional study, we therefore aimed to explore the relations between the perceived identity leadership of instructors of such group exercises classes and relevant outcomes. To this end, we surveyed 458 participants of Zumba classes in a university setting. More specifically, these participants rated their (experienced) instructors in terms of identity leadership and completed additional measures of their identification with the Zumba class, of different forms of motivation to participate, of their self-reported effort, and of their intention to attend to next sessions. The results revealed that the perceived identity leadership of the instructors was quite high, and was positively related to the identification with the Zumba class. In addition, identity leadership was positively related to both self-related effort during the class and the intention to attend the next class. These findings underline the relevance to further develop and train the identity leadership of group exercise leaders.

